# Transient increases in intracellular calcium and reactive oxygen species levels in TCam-2 cells exposed to microgravity

**DOI:** 10.1038/s41598-017-15935-z

**Published:** 2017-11-15

**Authors:** C. Morabito, S. Guarnieri, A. Catizone, C. Schiraldi, G. Ricci, M. A. Mariggiò

**Affiliations:** 1 Department of Neuroscience, Imaging and Clinical Sciences and Centro Scienze dell’ Invecchiamento e Medicina Traslazionale (CeSI-MeT), “G. d’Annunzio” University of Chieti-Pescara, Chieti, Italy; 2 Section of Histology and Medical Embryology, Department of Anatomy, Histology, Forensic and Orthopaedic Medicine, “Sapienza” University of Rome, Rome, Italy; 3 Department of Experimental Medicine, Università degli Studi della Campania “Luigi Vanvitelli”, Naples, Italy

## Abstract

The effects of microgravity on functions of the human body are well described, including alterations in the male and female reproductive systems. In the present study, TCam-2 cells, which are considered a good model of mitotically active male germ cells, were used to investigate intracellular signalling and cell metabolism during exposure to simulated microgravity, a condition that affects cell shape and cytoskeletal architecture. After a 24 hour exposure to simulated microgravity, TCam-2 cells showed 1) a decreased proliferation rate and a delay in cell cycle progression, 2) increased anaerobic metabolism accompanied by increased levels of intracellular Ca^2+^, reactive oxygen species and superoxide anion and modifications in mitochondrial morphology. Interestingly, all these events were transient and were no longer evident after 48 hours of exposure. The presence of antioxidants prevented not only the effects described above but also the modifications in cytoskeletal architecture and the activation of the autophagy process induced by simulated microgravity. In conclusion, in the TCam-2 cell model, simulated microgravity activated the oxidative machinery, triggering transient macroscopic cell events, such as a reduction in the proliferation rate, changes in cytoskeleton-driven shape and autophagy activation.

## Introduction

Over the last century, we have observed a sudden, ever-growing increase in the number of space flights not only for space exploration and the building/maintenance of satellites and space stations but also for space tourism and commercial space flights. Consequently, studies investigating the permanent effects of altered gravity on astronauts in space are required. Indeed, during space flight, possibly conflicting “environments” are present, including g-forces, launch-associated vibrations, exposure to microgravity for long periods, changes in cabin gases, and cosmic radiation. Thus, experimental models or adequate controls for all the different factors to which astronauts or space-flown animals are exposed are difficult to define. However, the main reproducible feature present in space is the weightless condition caused by microgravity, which alters physical processes in biological organisms. The effects of microgravity on the cardiovascular system and blood flow are well-known^1^, as are their effects on renal functions^2^. Other main target systems of microgravity include the musculo-skeletal apparatus^3,4^, branches of the somatic and autonomous nervous system^5,6^, and the endocrine system^7^. Microgravity also alters the reproductive system by influencing its specific functions and the associated endocrine signals^8–11^. In particular, *in vitro* and *in vivo* observations revealed that testicular function was impaired in response to microgravity exposure. Indeed, near weightless conditions affect cell proliferation, differentiation, germ cell survival, apoptosis, and the secretion of sexual hormones from testicles or testicular cell cultures^12–16^. These effects may be the cause and a partial explanation for post-flight dysfunction or dysfunction observed following exposure to simulated microgravity (s-microgravity). Moreover, the acute microgravity-induced alterations in the physiology of testicular cells may obscure the starting point of mechanisms that lead to long-lasting tumourigenic processes. Unfortunately, male germ cells are only able to be cultured for a few hours, because these cell types are not able to survive and develop without the support of sustentacular (Sertoli) cells. However, seminoma cells, even if they are derived from a malignant derivative of male germ cells, maintain the biochemical and morphological features of the primordial germ cells/gonocytes, allowing their use as a good model of mitotically active male germ cells^17,18^. For this reason, TCam-2 cells were recently selected to study the *in vitro* effect of s-microgravity. This cell line was established from a primary lesion of a left testicular seminoma from a 35-year-old male patient^19^. These cells have also been well characterized at the molecular and biochemical levels and show a readiness to respond to extracellular growth factors^20–25^. Exposure of TCam-2 cells to s-microgravity deeply affects cell shape and architecture and induces microtubule disorientation and an increase in the actin microfilament network that increased the cell width, together with a transient collapse of the mechano-sensing microvilli-like structures. These peculiar cytoskeletal modifications have been proposed to be related to the autophagy process, which is postulated to be an adaptive cell response to s-microgravity, likely allowing the cell to survive in a modified physical microenvironment^24^.

The aim of the present study was to investigate intracellular signalling and cell metabolism in TCam-2 cells exposed to s-microgravity to depict the intracellular status related to macroscopic cellular changes (such as cell architecture and shape, cell proliferation and cell cycle changes) induced by the modification of extracellular gravitational forces. This model may be useful for identifying possible protective strategies.

## Results

### Biological effects induced by s-microgravity

**Figure 1:**
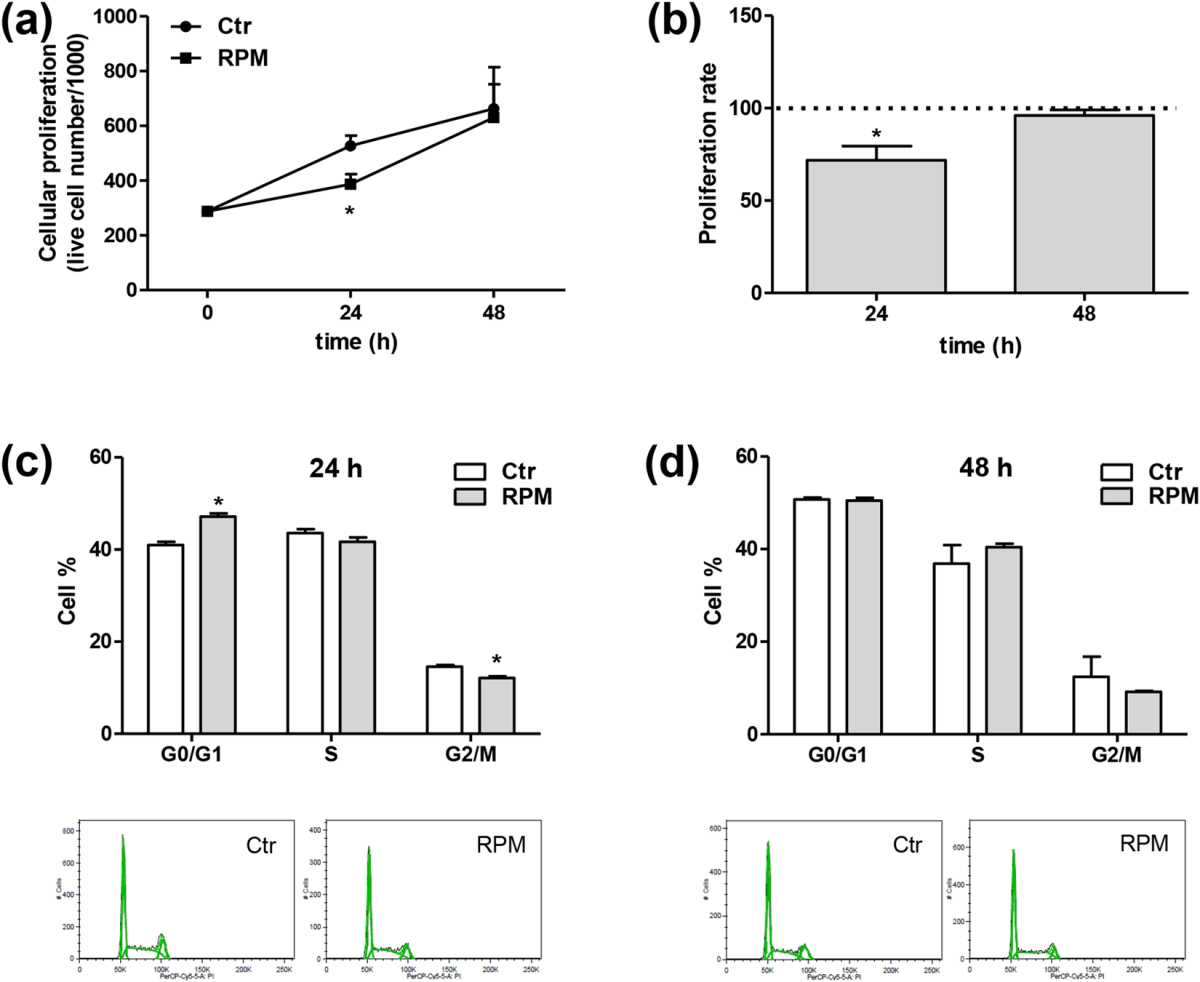
Effect of s-microgravity on cell growth and the cell cycle. (**a**) Cell growth under standard (at 1 g, Ctr) or s-microgravity (on the RPM) conditions. Values are presented as means ± SEM, n = 3. *p < 0.05 compared with the corresponding Ctr. (**b**) The proliferation rate is expressed as the percentage of live cells exposed to s-microgravity versus live control cells, which were set to 100%, at each time point (24 and 48 h). Values are presented as means ± SEM, n = 3. *p < 0.05 compared with the corresponding Ctr. (**c**) and (**d**) The percentage of cells in different phases of the cell cycle under standard (at 1 g, Ctr) or s-microgravity (RPM) conditions. Values are presented as means ± SEM, n = 3. *p < 0.05. The graphs under the histograms are representative cell cycle profiles of Ctr and RPM cells cultured for 24 or 48 h.

TCam-2 cells were exposed to s-microgravity using a random positioning machine (RPM) for up to 48 hours, a time interval that was useful for observing acute effects and was coherent with cell cycle, which was approximately 24–36 hours for the cell density used in this experiment. All results obtained from exposed cells were compared to those from cells grown at 1 g (control cells) in the same CO_2_ incubator. Data obtained by monitoring cell proliferation and the cell cycle at 24 or 48 hours of exposure revealed a transient delay in cell growth at the 24 h time point, with a concomitant decrease in the cell proliferation rate (Fig. 1a and b). At the 24 h time point, the inhibitory effect on cell proliferation was accompanied by a slight but significant increase in the percentage of cells in G0/G1 phase and a corresponding decrease in the percentage of cells in G2/M phase (Fig. 1c). After 48 h of exposure to s-microgravity, the distribution of cells among cell cycle phases was similar to the corresponding control cell population (Fig. 1d). Based on the results of trypan blue exclusion assays and cytofluorimetric analyses, the number of non-viable cells was similar between control and s-microgravity-exposed cells and remained between 5% and 10% of the total cells.

**Figure 2:**
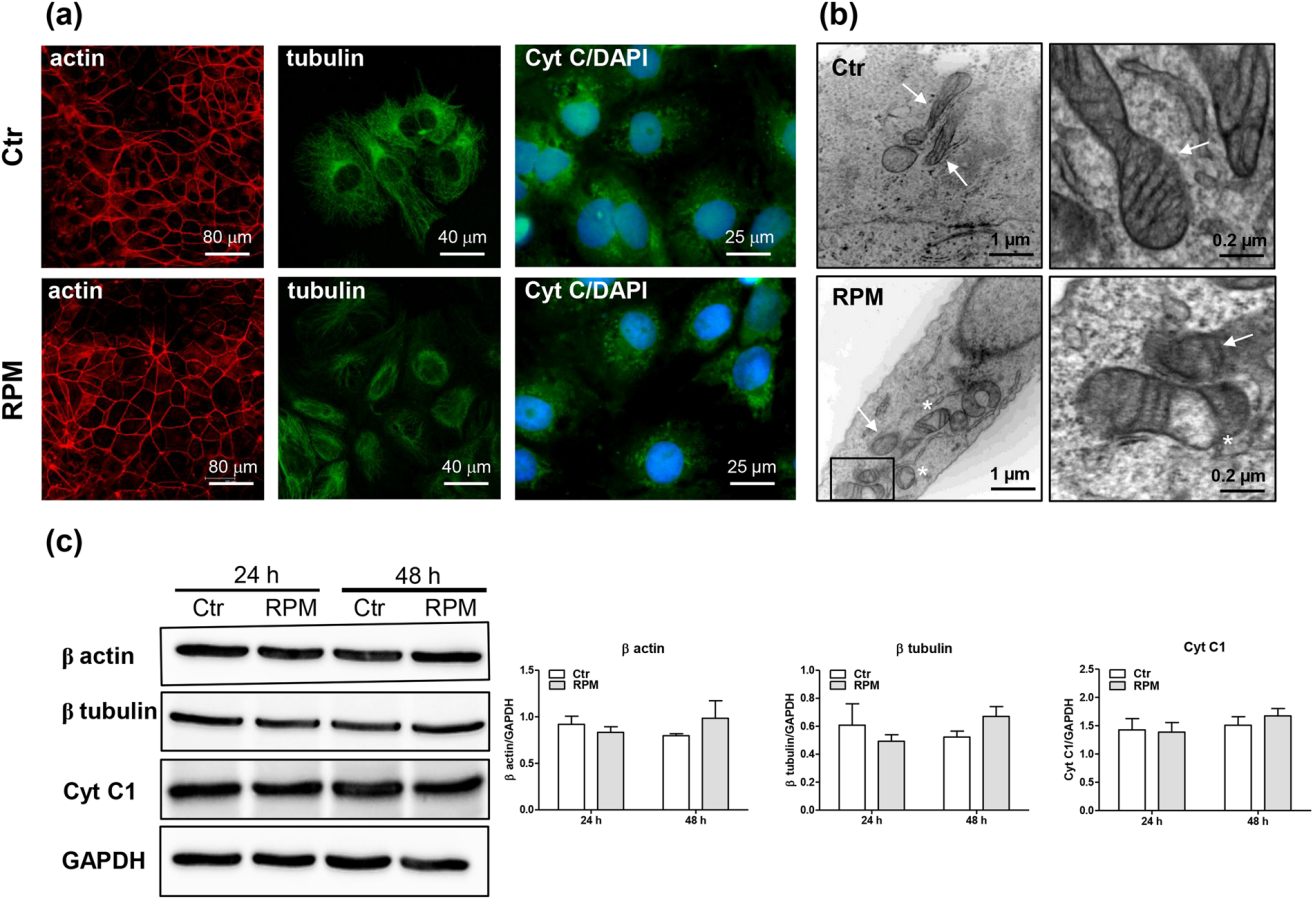
Changes in the cytoskeleton and mitochondria in cells exposed to microgravity. (**a**) Representative images of cells grown under standard (at 1 g, Ctr) or s-microgravity (on the RPM) conditions and stained as described in Methods. (**b**) Ultrastructural analysis of cells cultured for 24 hours under Ctr or RPM conditions. White arrows indicate normal-shaped mitochondria, white asterisks indicate altered mitochondria. The box, in RPM image, indicates the magnified view shown in the side panel. (**c**) Representative immunoblots of actin, tubulin and cytochrome C (CytC1) expression levels and the corresponding densitometric analyses. In particular, the representative blots shown here are derived from the same membrane probed for actin and GAPDH and another membrane probed for tubulin and CytC1. The densitometric analyses are plotted as the relative expression calculated as the ratio between the optical density (OD) × mm^2^ of each band and OD × mm^2^ of the corresponding GAPDH band, which was used as the loading control, probed on the same membrane after stripping (stripping was performed according to the instructions provided by the manufacturer of the nitrocellulose membranes, Protran; Whatman-GE Healthcare). The relevant proteins were detected using chemiluminescence kits (Pierce EuroClone S.p.A., Pero, Italy), and signals were acquired and analysed using an image acquisition system (Uvitec mod Alliance 9.7, Uvitec, Cambridge, UK); densitometric data were plotted using Prism5 software (GraphPad, San Diego, CA, USA). In the densitometric analyses, the data are presented as the means ± SEM from three independent experiments.

The alteration in external forces caused by s-microgravity conditions also induced transient modifications in cell shape and architecture. Consistent with a previous report^26^, 24 h of exposure to s-microgravity affected the cytoskeleton scaffold in TCam-2 cells. Indeed, tubulin localization primarily appeared to be de-structured in cells exposed to s-microgravity for 24 hours. In particular, as previously described^26^, centriolar polarization was impaired, and microtubules appeared caotically distributed. In addition, we did not observe any difference in the expression levels of the main protein components of the cytoskeleton (Fig. 2a and c).

After 48 h of exposure, the tubulin organization resembled control cells (Fig. S1a).

The expression and localization of cytochrome C did not change in the exposed cells at either 24 or 48 h compared to those observed in control cells (Fig. 2a and c).

Notably, the ultrastructural analysis revealed a transient modification in the morphology of mitochondria in cells exposed to s-microgravity for 24 hours. In particular, in these cells, the mitochondria appeared swollen and their inner matrix appeared filled with a non-electron-dense (Fig. 2b). Quantitative analyses performed on at least 25 images of control and exposed cells, at the same magnification, revealed that the altered mitochondria were 3.1 ± 1.05% of the total mitochondria present in the field of control cells, whereas the altered mitochondria were 42.1 ± 10.75% of the total mitochondria present in the field of s-microgravity-exposed cells. After 48 hours of s-microgravity-exposure, the mitochondrial morphology appeared similar to the corresponding control cells (Fig. S1b).

### Intracellular responses triggered by s-microgravity exposure

**Figure 3:**
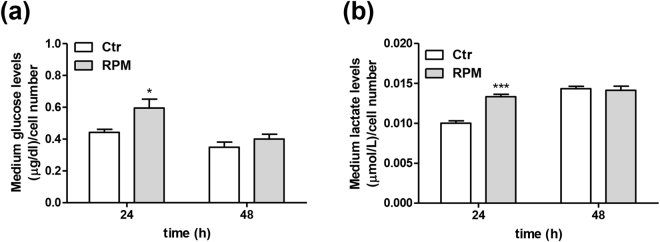
Cellular metabolism after exposure to s-microgravity. (**a**) Glucose levels were measured in the growth medium of cells grown at 1 g (Ctr) or exposed to s-microgravity (RPM) and are expressed as the ratio between glucose levels and the number of cells at each time point. Values are presented as means ± SEM, n = 3. *p < 0.05 compared with the corresponding Ctr. (**b**) Lactate levels were measured in cell medium from cells grown under the same conditions as described in (a) and are expressed as the ratio between lactate levels and the number of cells at each time point. Values are presented as means ± SEM, n = 3. ***p < 0.001 compared with the corresponding Ctr.

The s-microgravity-induced modifications in TCam-2 cell proliferation and cell cycle distribution, as well as in their morphology, led us to verify whether and how metabolic activity and certain intracellular signals involved in many cell functions may be affected. Glucose and lactate levels in the cell medium were measured to obtain an overall perspective of the cellular metabolic activity. Glucose and lactate levels were significantly increased in growth medium from cells exposed to s-microgravity for 24 hours compared to the levels in medium from the corresponding control cells (Fig. 3a and b, respectively). In addition, this effect was confined within the 24 h exposure window; indeed, after 48 h of exposure, no differences in glucose and lactate levels were observed between s-microgravity-exposed cells and control cells (Fig. 3a and b, respectively).

**Figure 4:**
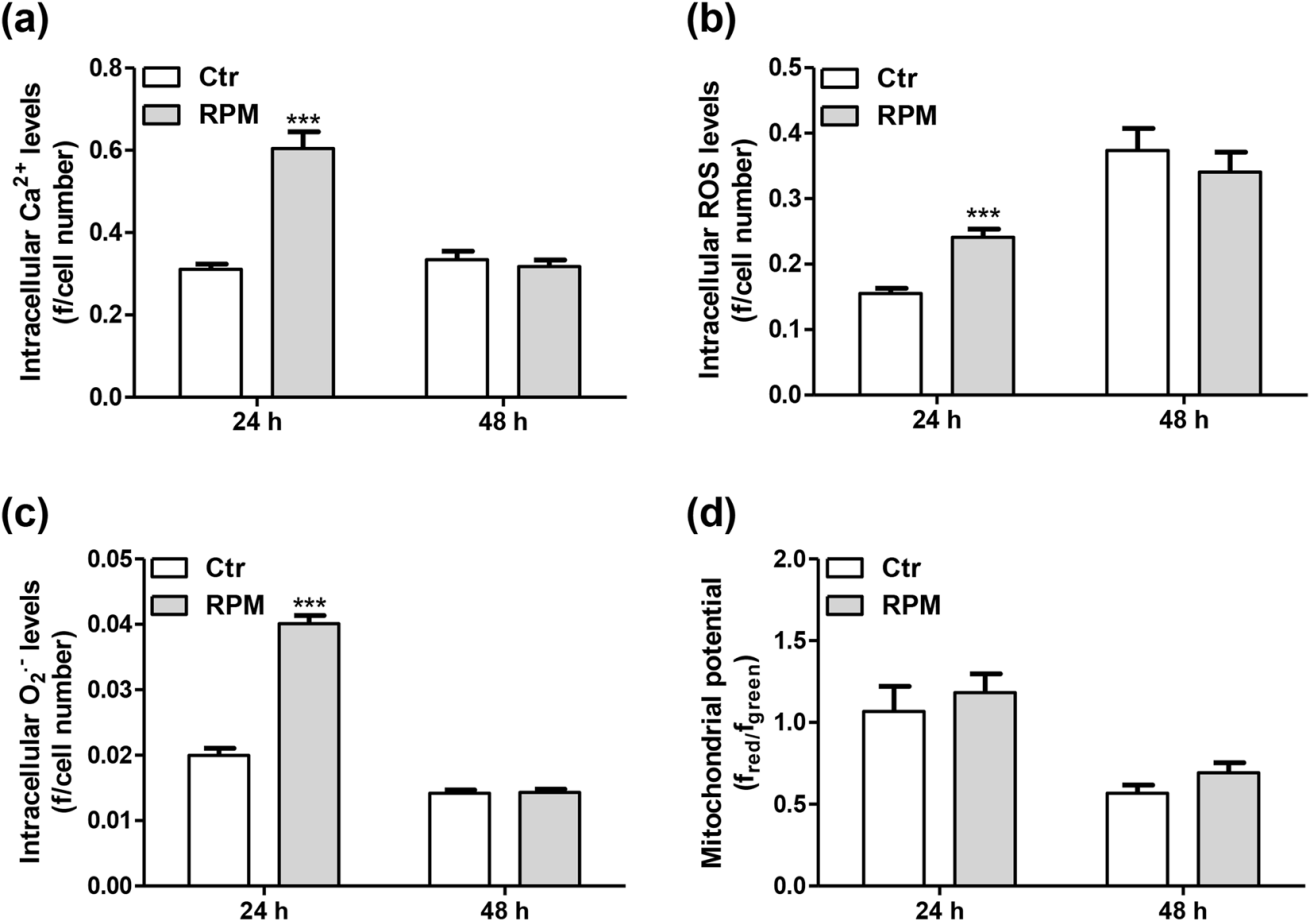
Cellular metabolism after exposure to s-microgravity. (**a**–**c**) Intracellular Ca^2+^, ROS and O_2_^•−^ levels, respectively, in live cells grown at 1 g (Ctr) or exposed to s-microgravity (RPM); values are expressed as the ratio between fluorescence emission (**f**) and the number of loaded cells at each time point. Values are presented as means ± SEM, n = 5. ***p < 0.001 compared with the corresponding Ctr. (**d**) Mitochondrial membrane potential in live cells grown under the same conditions described in (**a**–**c**); values are expressed as the ratio between red and green fluorescence at each time point. Values are presented as means ± SEM, n = 8. ***p < 0.001 compared with the corresponding Ctr.

Measurements of intracellular Ca^2+^ levels, the production of extremely reactive oxygen molecules (i.e., O_2_^•−^ and reactive oxygen species (ROS)) and mitochondrial function (by monitoring the mitochondrial membrane potential) were performed to examine cellular activity. Interestingly, increased levels of Ca^2+^, ROS and O_2_^•−^ were detected in cells exposed to s-microgravity for 24 h compared to the levels in the corresponding control cells (Fig. 4a,b and c, respectively). These effects were not detectable in the cells after 48 h of exposure (Fig. 4a, b and c). The mitochondrial membrane potential did not change significantly after exposure (Fig. 4d).

### Strategies to counteract s-microgravity-induced effects

**Figure 5:**
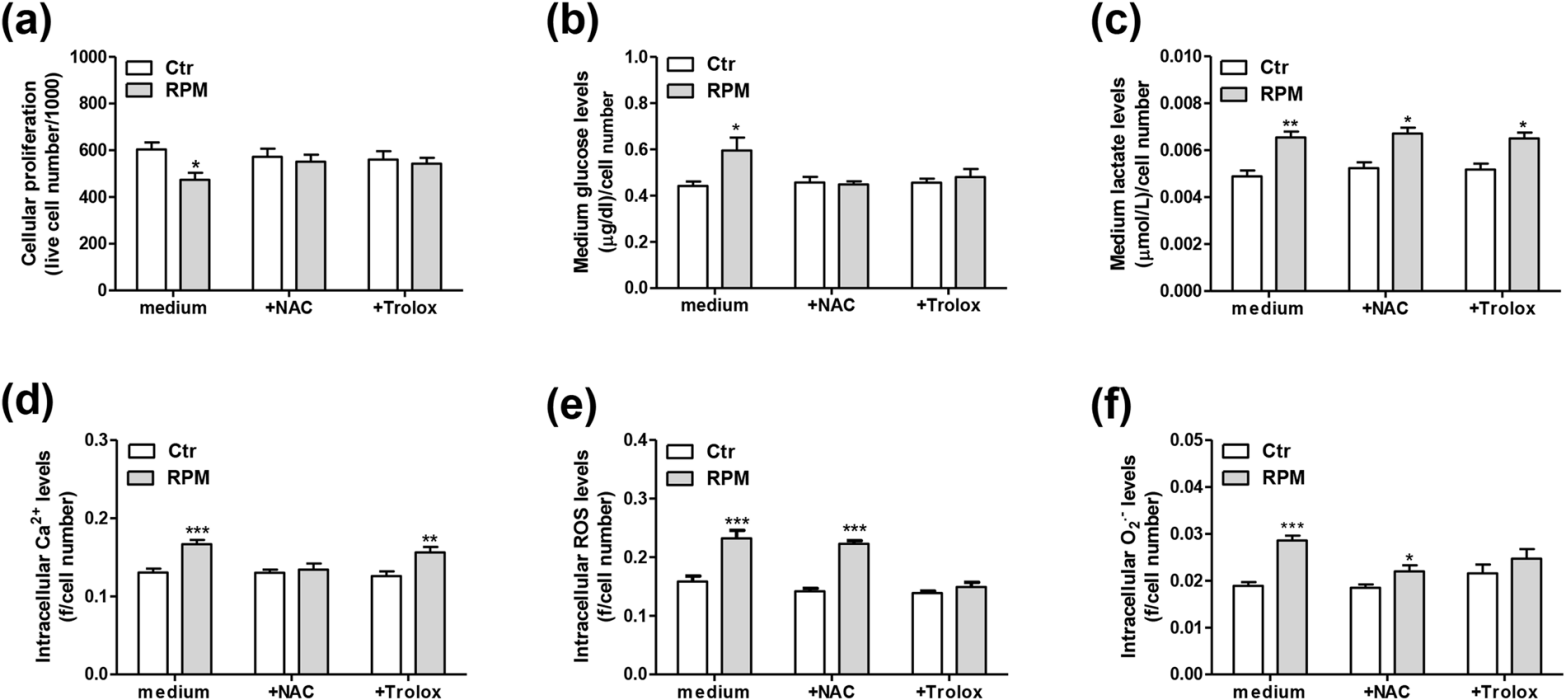
Antioxidants counteract the effects of s-microgravity. Results were obtained from cells incubated with growth medium (medium) or medium containing NAC (+NAC) or Trolox (+Trolox) under standard (at 1 g, Ctr) or s-microgravity (RPM) conditions for 24 h. Cell numbers (**a**), glucose levels (**b**) and lactate levels (**c**) are expressed as means ± SEM, n = 3. *p < 0.05, **p < 0.01 compared with the corresponding Ctr. Ca^2+^ (**d**), ROS (**e**), and O_2_^•−^ (**f**) levels are expressed as means ± SEM, n = 5. *p < 0.05, **p < 0.01, ***p < 0.001 compared with the corresponding Ctr.

Based on the results described above, the presence of s-microgravity conditions altered the oxidative balance of TCam-2 cells. We examined cell behaviour after a 24 h incubation with two anti-oxidant molecules, N-acetyl-cysteine (NAC) and 6-hydroxy-2,5,7,8-tetramethylchroman-2-carboxylic acid (Trolox), to determine whether we could establish conditions that prevent not only the oxidative imbalance but also the cellular response that are likely induced by oxidative stress. Both compounds counteracted the effects of s-microgravity on cell proliferation and glucose consumption. Indeed, the number of cells and the levels of glucose in their medium did not change in s-microgravity-exposed or control cells cultured with NAC or Trolox (Fig. 5a and b). Moreover, neither of the molecules modified the s-microgravity-induced effects on lactate production (Fig. 5c). Specifically, NAC counteracted the s-microgravity-induced effects on Ca^2+^ levels (Fig. 5d), whereas Trolox counteracted the s-microgravity-induced effects on ROS and O_2_^•−^ levels (Fig. 5e and f).

**Figure 6:**
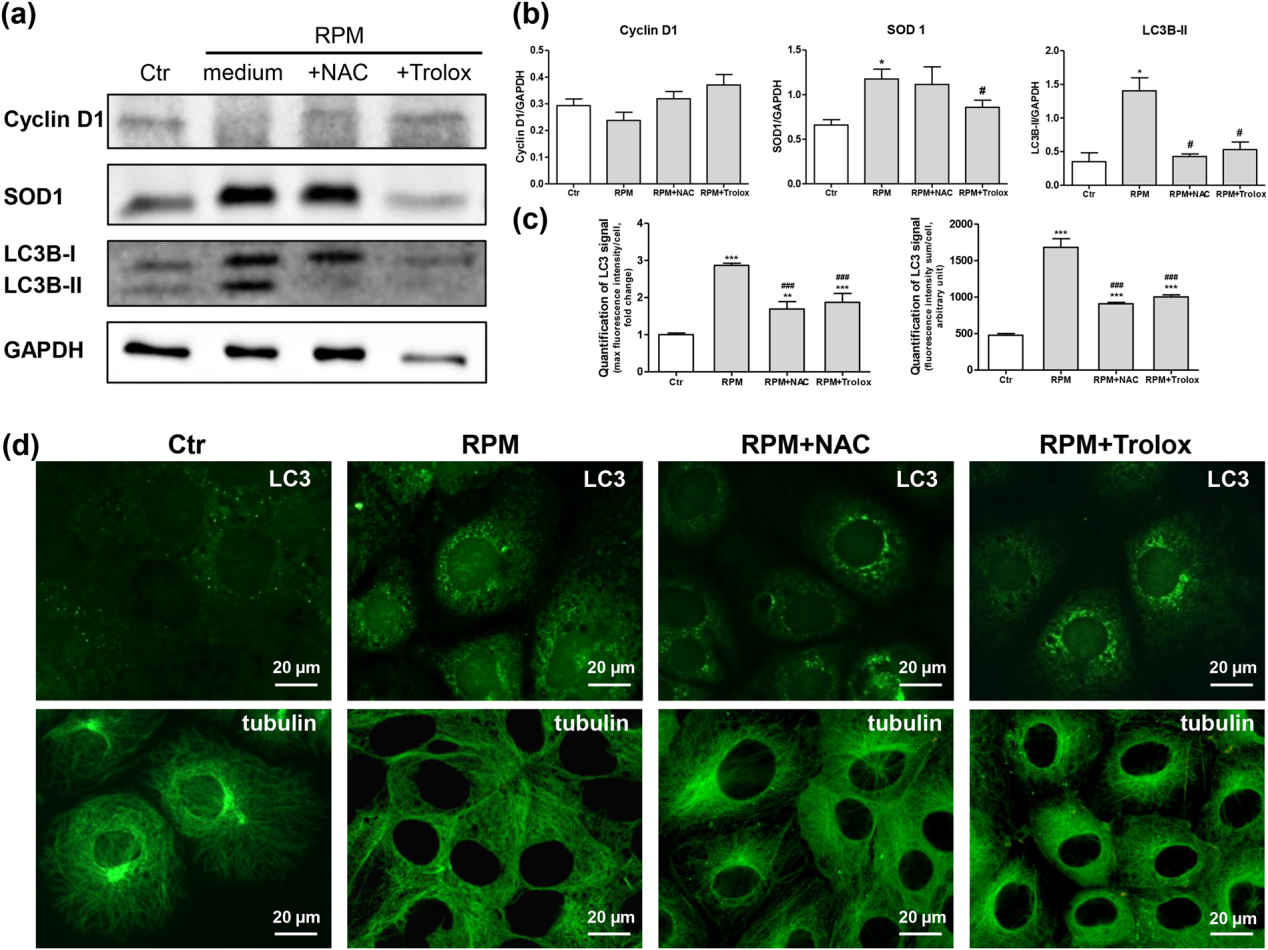
Antioxidants counteract the s-microgravity-induced expression of markers associated with autophagy and microtubule disorientation. (**a**,**b**) Representative images of Western blot analyses of cyclin D1, SOD1 and LC3 expression levels and densitometric analyses. In particular, the representative blots shown here are derived from the same membrane probed for cyclin D1 and SOD1 and another membrane probed for LC3 and GAPDH. The densitometric analyses are plotted as relative expression calculated as the ratio between the OD × mm^2^ of each band and OD × mm^2^ of the corresponding GAPDH band, which was used as the loading control, probed on the same membrane after stripping (stripping was performed according to the instructions provided by the manufacturer of the nitrocellulose membranes, Protran; Whatman-GE Healthcare). Relevant proteins were detected using chemiluminescence kits (Pierce EuroClone S.p.A., Pero, Italy), and signals were acquired and analysed using an image acquisition system (Uvitec mod Alliance 9.7, Uvitec, Cambridge, UK); densitometric data were plotted using Prism5 software (GraphPad, San Diego, CA, USA). In the densitometric analyses, the data are presented as the means ± SEM from three independent experiments. *p < 0.05 compared with the corresponding Ctr; ^#^p < 0.05 compared with cells grown under RPM conditions. (**c**) Graphs representing the “Maximum fluorescence amplitude”/cell and the “Sum of fluorescence intensity”/cell of anti-LC3 immunofluorescence images. Values were obtained using Leica Confocal software and are presented as the means ± SEM of 30 randomly selected fields/sample. **p < 0.01, ***p < 0.001 compared with the corresponding Ctr; ^###^p < 0.001 compared with cells cultured under RPM conditions. (**d**) The upper row shows representative images of immunofluorescence staining for LC3 in TCam-2 cells cultured under control conditions (Ctr) or exposed to s-microgravity without (RPM) or with NAC (RPM + NAC) or Trolox (RPM + Trolox) for 24 h. The lower row shows representative images of immunofluorescence staining for α-tubulin in TCam-2 cells grown under the conditions indicated above.

The presence of the antioxidants at least partially prevented the effects of s-microgravity on the expression levels of markers of the G1/S cell cycle transition (cyclin D1), oxidative metabolism (SOD1) and autophagy (LC3). Cyclin D1 levels appeared to decrease, although the difference was not significant, after 24 h of exposure to s-microgravity, whereas the levels were similar to control cells when NAC or Trolox were present in the medium (Fig. 6a and b). SOD1 expression appeared to increase in exposed cells, and the antioxidants partially or totally reversed this effect (Fig. 6a and b). Consistent with a previous report^26^, s-microgravity transiently induced autophagy in TCam-2 cells, and this microgravity-dependent autophagy activation has been proposed to be related to microgravity-induced microtubule disorientation. Interestingly, NAC and Trolox seemed to at least partially counteract the s-microgravity-induced autophagy. NAC and Trolox induced a quantitative decrease in LC3 protein levels, as revealed by the Western blot analysis, compared with the levels observed in cells exposed to s-microgravity alone (Fig. 6a and b). Interestingly, the LC3 fluorescence signal was very low in some cells that had been exposed to s-microgravity in the presence of NAC or Trolox for 24 hours, exhibiting a pattern similar to control cells (Fig. 6d). This qualitative observation was confirmed by quantitative analyses of immunofluorescence staining. The highest and sum fluorescence intensities of LC3-stained cells increased in images of cell samples exposed to s-microgravity for 24 hours compared with those in images of control cells, whereas these parameters were decreased in samples exposed to s-microgravity in the presence of NAC or Trolox compared with those in samples exposed to growth medium alone (Fig. 6c). Similar results were obtained when we analysed regions of interest (ROIs) in a representative experiment. The stack profiles of representative ROIs revealed that the maximum amplitude of the fluorescent signal was reduced in the presence of antioxidants compared with the ROIs observed in samples cultured on the RPM in growth medium alone (Fig. S2). In addition, tubulin assembly, which was altered by s-microgravity exposure, was recovered in the presence of the antioxidants. Indeed,the tubulin localization in cells exposed to s-microgravity for 24 h and incubated with NAC or Trolox resembled control cells (Fig. 6d).

## Discussion

Testicular germ cell tumours originate from embryonic germ cells (primordial germ cells or gonocytes) that are converted into a pre-invasive lesion, known as germ cell neoplasia *in situ*. The initiation of this neoplastic transformation is most likely triggered by a disruption in the local embryonic niche that results in delayed or blocked germ cell differentiation^27,28^. Within an average of 9 years, germ cell neoplasia *in situ* produces Type II testicular germ cell tumour cells: seminomas and non-seminomas. Among these types of tumours, seminomas are considered the default developmental pathway of germ cell neoplasia *in situ* cells and maintain most of the molecular and biochemical features of foetal germ cells. The importance of the microenvironment for cell behaviour and, consequently, the possible onset, development and progression of seminoma lesions has been highlighted in several studies^29^. In addition, the TCam-2 cell line, which is, as previously mentioned, a seminoma cell model, displays extraordinary versatility and plasticity. Indeed, TCam-2 cells differentiate into germ cell neoplasia *in situ*/seminoma lesions when transplanted orthotopically in seminiferous tubules, whereas the same cells adopt an embryonal carcinoma-like fate when grafted into the flank or corpus striatum of a host animal^30^. Notably, different host tissues provide different molecular cues and different physical forces that act on the microenvironment in terms of the stiffness to which the cells are subjected. The microenvironment-dependent behaviour of TCam-2 cells makes this cellular model particularly suitable for studying the mechanisms by which external physical forces influence cell behaviour. Indeed, the presence of s-microgravity conditions was recently shown to induce significant cytoskeletal remodelling without altering cell proliferation, as tested by determining the percentage of cells expressing phosphorylated histone H3^26^. H3 phosphorylation is usually associated with the early mitotic phase because its activation is required to initiate mitotic chromosome segregation and condensation^31^. These previously reported results are consistent with the findings of the present study. In proliferating TCam-2 cells exposed to s-microgravity for up to 48 hours, we observed a delay in growth after approximately 24 hours that was recovered at 48 hours, without resulting in a significant percentage of dead cells. This delay was caused by transient accumulation of a small but significant percentage of cells in G1 phase, likely due to the s-microgravity-induced effects on cytoskeletal arrangement. However, DNA synthesis did not appear to be affected because the percentage of cells in S phase was not different from cells grown under 1 g conditions. The cell cycle distribution and the size of the effect may explain its transient timing without a particular acceleration in the proliferation rate, thus allowing the recovery of a number of cells in samples exposed to s-microgravity for 48 hours. Other studies showed contrasting results with regard to the effects induced by s-microgravity, which was reported to not affect the viability and proliferation of primary mouse Sertoli cells^32^, to exert beneficial effects on both the survival of testis germ cells^33^ and the proliferation of spermatogonial stem cells^34^, and to inhibit cell proliferation and cycle progression in mesenchymal stem cells^35–37^ compared to the respective control cultures. These results are at least partially inconsistent with the findings of the present study, but most of the previous studies were performed using a rotary cell culture bioreactor that allows cells to aggregate and maintains cells in suspension conditions that are postulated to provide better nutrient and oxygen supply for cultured cells than control cells, even if this benefit was denied to inner cells in the aggregates^33^. However, in the present study, we used an RPM to simulate microgravity conditions, preserving one of the main characteristics of TCam-2 cells, cell adhesion to a substrate. The use of an RPM to simulate microgravity conditions has also been shown to inhibit cell proliferation in other cell models, such as the lung cancer H1703 cell line^38^, FTC-133 thyroid cancer cells^39^, and MC3T3-E1 murine osteoblasts^40^, depending on the exposure time.

In addition, the contradictory data regarding the effects of s-microgravity on cell proliferation depend on the substantial heterogeneity of tested cells and indicate that s-microgravity itself promotes the emergence of distinct phenotypes (recognizable by differences in shape, biochemical pathways, and behavioural processes)^40,41^. This finding highlights the importance of contextualizing the results in regard to the simulation model used.

More concordant results depict the effect of s-microgravity on tissue and cell structures. Indeed, according to the results of *ex vivo* and *in vitro* experiments, the testis cords appeared de-structured^33,42^ and cytoskeleton disorganization became evident during s-microgravity exposure^43–47^. As expected and consistent with previously published results^26^, transient microtubule disorganization was evident in our cell model after 24 h of exposure to s-microgravity. Interestingly, this disorganization was accompanied by changes in the morphology of mitochondria, which appeared swollen, with an inner matrix that exhibited a different electron density to that of control cells. We hypothesize that these changes may reflect the metabolic status of these cells. Indeed, TCam-2 cells exposed to s-microgravity for 24 hours exhibited reduced uptake of extracellular glucose and increased lactate production, indicating increased anaerobic metabolism. This increase in metabolism was accompanied by an increase in Ca^2+^ and ROS levels, particularly species produced by mitochondrial activity, without modifying the mitochondrial potential. The presence of an altered metabolic condition and/or oxidative stress and mitochondrial dysfunction has been documented in many *in vivo* and *in vitro* models exposed to real or simulated microgravity. The presence of many oxidative stress markers has been reported in blood from cosmonauts and pilots experiencing routine or short-term gravitational changes^48,49^. Similar effects on an increase in ROS production and metabolic unbalance were observed *in vivo* and *in vitro* in different models subjected to s-microgravity conditions. For example, an increase in ROS production in cerebral but not mesenteric smooth muscle cells, profound effects on the levels of metabolic proteins in the hippocampus, and cerebrovascular mitochondrial dysfunction were observed in a rat tail suspension model^50–52^. An increase in ROS production was also observed in some cell types, such as neuron-like cells and fibroblasts^53–55^.

In our model, the increase in ROS production appears to be responsible for the biological effects induced by s-microgravity exposure. The Ca^2+^/ROS interplay is broadly considered an intracellular signal that decodes the cellular metabolic status and cellular response to external stimuli^56,57^. In our model, the s-microgravity-induced increase in ROS levels after 24 h of exposure was responsible for the reduced extracellular glucose uptake, changes in cell shape, delay in cell proliferation and activation of autophagy processes. Indeed, all these effects were prevented when cells were cultured with the antioxidant molecules NAC or Trolox in the extracellular medium. However, the metabolic status of TCam-2 cells under s-microgravity conditions and in the presence of antioxidants cannot be simplified as described above because neither molecule prevented the s-microgravity-induced increase in lactate production, and only Trolox completely prevented the increase in O_2_^•−^ and ROS levels (with a partial counteracting effect on the increase in intracellular Ca^2+^ levels). Meanwhile, NAC only partially blocked the increase in O_2_^•−^ and ROS levels. In any case, although the effects of s-microgravity on TCam-2 cells *in vitro* appeared to be transient, a possible long-lasting hidden effect cannot be excluded, considering that this external stimulus affected cell metabolism and particularly mitochondrial functions, which are extremely important for cell health, specifically sperm cells and their precursor cells^58^.

In conclusion, in the TCam-2 cell model, s-microgravity activated the oxidative machinery that, on one hand, induced macroscopic cell events, such as a reduction in the proliferation rate, changes in cytoskeleton-driven shape and activation of some autophagy pathways; on the other hand, this intracellular signalling also triggered an adaptive response that caused transient effects after 24 h of exposure. Notably, antioxidants did not prevent the increase in lactate production triggered by s-microgravity conditions, indicating that this event did not depend on ROS production. This evidence provides a new perspective for further investigations, which, in addition to increasing our knowledge about the effects of microgravity, may have repercussions on our understanding of tumour cell metabolism.

## Methods

### Equipment and cell exposure parameters

Microgravity conditions were simulated using an RPM connected to a control console through standard electrical cables (Dutch Space, Leiden, The Netherlands). The apparatus is a 3D clinostat consisting of two independently rotating frames. One frame is positioned inside the other, exerting a very complex net change in orientation on a biological sample mounted in the middle. The degree of microgravity simulation depends on the angular speed and the inclination of the disk. This apparatus does not actually eliminate gravity, but the RPM is a microweight simulator based on the principle of “gravity-vector averaging”: it allows a 1 g stimulus to be applied omnidirectionally rather than unidirectionally, and the sum of the gravitational force vectors tends to equal zero. The effects generated by the RPM are comparable to the effects of real microgravity, provided that the direction changes are faster than the response time of the system to the gravity field. The desktop RPM we used was positioned within an incubator (to maintain the 37 °C temperature and CO_2_ and humidity levels).

### Chemicals and materials

Unless indicated otherwise, the cell culture medium, sera, and antibiotics were purchased from ThermoFisher (Monza, Italy), the cell culture plasticware was obtained from Becton Dickinson Falcon (Steroglass S.r.l., San Martino in Campo, Italy), and the reagents and standards were obtained from Sigma-Aldrich (Milan, Italy).

### Cell cultures

The human TCam-2 cell line (kindly provided by Prof. Claudio Sette, Tor Vergata University, Rome, Italy) was cultured in growth medium (RPMI 1640 supplemented with 10% foetal bovine serum and penicillin/streptomycin) at a density of 30 × 10^3^ cells/cm^2^. Twenty-four hours after cells were seeded, experiments were performed on cells cultured for 24 and 48 hours at 1 g in the same incubator containing the RPM or at s-microgravity in the RPM. For MTT assays, measurements of Ca^2+^, ROS, and O_2_^•−^ levels, and mitochondrial membrane potential measurements, TCam-2 cells were plated in special-optic 96-well plates (Corning-Costar, Milan, Italy). For trypan blue exclusion tests, cytofluorimetric analyses, Western blots, analyses of glucose and lactate levels, and transmission electron microscopy (TEM) analyses, cells were plated in 35 mm Petri dishes. For immunofluorescence staining, cells were plated on glass slides or IBIDI microscopy chambers (IBIDI GmbH, Martinsried, Germany). All cell culture holders (microplates, dishes, etc.) were completely filled with culture medium and placed in both 1 g and RPM culture conditions to avoid air bubbles and to minimize liquid flow;thus, the effects of both buoyancy and shear stress during rotation were negligible.

### Viability and proliferation assays

The trypan blue exclusion assay was performed by staining the cells with a trypan blue dye solution (0.5% in phosphate-buffered saline, PBS), and the stained cells were counted using a Bürker chamber. Blue-stained cells were considered non-viable. Cell growth was tested using a colorimetric assay based on reduction of 3-(4,5-dimethylthiazol-2-yl)-2,5-diphenyltetrazolium bromide (MTT). At the selected times, MTT was added to each well (0.5 mg/mL final concentration). After a 3 h incubation at 37 °C, the medium was removed and dimethyl sulfoxide (200 μL) was added to each well. After a 30 min incubation at 37 °C, the absorbance of each well was read at 560 nm using a microplate reader (SpectraMAX 190, Molecular Devices, Toronto, ON, Canada), and the number of cells was determined using a calibration curve.

### Flow cytometry analysis

Cell cycle investigations were performed according to published protocols^59^. Cells were washed with PBS, harvested with trypsin/EDTA, and centrifuged (500xg, 5 min). Cell pellets were fixed with 70% (v/v) ethanol and stained with a propidium iodide solution (50 µg/mL propidium iodide and 100 µg/mL RNAse in PBS, Sigma-Aldrich). The fluorescent intensity was recorded using a FACS Calibur flow cytometer (Becton Dickinson Biosciences, Franklin Lakes, NJ, USA). Samples were cultured in triplicate, and a minimum of 10,000 events were acquired for each pellet. The percentages of cells in G0/G1, S, and G2/M phases of the cell cycle, as well as apoptotic and necrotic populations were calculated using the ModFit program (Becton Dickinson Biosciences).

### Immunofluorescence staining

Cells were fixed with 4% paraformaldehyde in PBS for 10 min at 4 °C and permeabilized with 1% bovine serum albumin and 0.1%Triton X-100 in PBS for 1 h at room temperature. Cells were incubated with the following primary antibodies diluted in PBS containing 1% BSA/0.1% Triton X-100 overnight at 4 °C: mouse monoclonal anti-α-tubulin antibody (1:75 dilution, Biomeda, Foster City, CA, USA), mouse monoclonal anti-cytochrome C1 antibody (1:10 dilution, Santa Cruz Biotechnology, Heidelberg, Germany), and rabbit polyclonal anti-LC3 antibody (1:120 dilution, Sigma-Aldrich). After rinsing, samples were incubated with a specific secondary antibody diluted in PBS for 90 min at room temperature (1:200 dilution, FITC-conjugated donkey anti-rabbit or anti-mouse IgGs or TRITC-conjugated donkey anti-mouse IgGs, Jackson ImmunoResearch, Newmarket Suffolk, UK). Then, samples were washed and mounted in buffered glycerol (0.1 M, pH 9.5).

Cells were fixed with 4% paraformaldehyde in PBS for 10 min at 4 °C, and permeabilized with cold ethanol:acetone (1:1) for 10 min at 4 °C to label F-actin. After rinsing, cells were incubated with rhodamine-conjugated phalloidin (1:40 dilution, Invitrogen Molecular Probes, Eugene, OR, USA) for 25 min, washed in PBS, and mounted.

Images of each sample were acquired using a Zeiss fluorescence microscope (Axioscope) and a Leica confocal microscope (Laser Scanning TCS SP2) equipped with Kr/Ar and He/Ne lasers.

Optical spatial series (23/25 optical sections with a step size of 2 μm) were captured to quantitatively analyse LC3 fluorescence. The intensity of the fluorescent LC3 signal was determined with Leica confocal software using the following parameters: the maximum amplitude of fluorescence (MAX amplitude), representing the maximum fluorescence intensity of each series; and the sum of the intensity, representing the total fluorescent intensity recovered within the z-axis of each series. The quantitative data from each series were normalized to the number of cells in each stack profile. The MAX amplitude in equivalently sized regions (ROIs) that comprised the nuclear and peri-nuclear area of the cells in each stack profile was also analysed.

### TEM analysis

Cells were fixed with 2.5% glutaraldehyde in 0.1 M cacodylate buffer (pH 7.4) for at least 1 h, post-fixed with 1% OsO_4_ in 0.1 M cacodylate buffer, mechanically and gently detached, centrifuged, de-hydrated in ethanol, and embedded in epoxy resin. Ultrathin sections (60 nm) were treated with tannic acid and then contrasted with lead hydroxide. Images were acquired using a Libra 120 transmission electron microscope (Zeiss) equipped with a wide-angle dual-speed CCD camera sharp: eye 2 K (4Mpx) operated by iTEM software (Soft Image System, Münster, Germany).

### Western blotting

Cells were scraped, lysed, and collected in sample buffer (62.5 mM Tris-HCl, pH 6.8, 2% SDS, 10% glycerol, 0.1 M dithiothreitol, and 0.002% bromophenol blue). Protein concentrations were determined using a protein assay kit (Bio-Rad DC; Bio-Rad, Segrate, Italy). Cell extracts were separated on 7.5% or 10% (w/v) homogeneous slab gels (40 μg of protein/lane) using SDS-PAGE and then transferred to nitrocellulose membranes (Protran; Whatman-GE Healthcare, Milan, Italy). Membranes were hybridized with a rabbit polyclonal anti-LC3 antibody (1:1,000 dilution, Sigma-Aldrich), mouse monoclonal anti-cytochrome C1 antibody (1:10 dilution, Santa Cruz Biotechnology), mouse monoclonal anti-beta tubulin or beta actin antibody (1:1,000 dilution, Santa Cruz Biotechnology) followed by an incubation with horseradish-peroxidase-conjugated anti-mouse or anti-rabbit IgGs (1:10,000 dilution, GE Healthcare, Cologno Monzese, Italy). The relevant proteins were detected using chemiluminescence kits (Pierce EuroClone S.p.A., Pero, Italy), and the signals were acquired and analysed using an image acquisition system (Uvitec mod Alliance 9.7, Uvitec, Cambridge, UK). A mouse monoclonal anti-GAPDH antibody (1:5,000 dilution, Merck S.p.a., Vimodrone, Italy) was used as a loading control.

### Measurements of glucose and lactate levels in cell culture medium

Glucose and lactate levels in the growth medium were detected using a Free Style Optium glucometer (Abbot Laboratories, Rome, Italy) and a Lactate Pro Analyser (Arkray Inc. Kyoto, Japan), respectively.

### Spectrofluorimetric measurements

Cells were incubated with a normal external solution (NES: 140 mM NaCl, 2.8 mM KCl, 2 mM CaCl_2_, 2 mM MgCl_2_, 10 mM glucose and 10 mM HEPES, pH 7.3) containing one of the probes reported in Table 1 (Thermo Fisher Scientific) for 40 min at 37 °C. After the sample was rinsed, fluorescence was detected using a microplate reader (SpectraMax Gemini XS; Molecular Devices) at 25 °C. Excitation and emission filters were set according to the probe used (Tab. 1).

**Table 1:** Fluorescence probes used for intracellular analyses.

Probe	Excitation (nm)	Emission (nm)	Analyses
Fluo4 –AM 5 µM	488	520	Intracellular Ca^2+^ levels
H_2_-DCFDA 10 µM	488	520	Intracellular ROS levels
MitoSox RED 5 µM	510	580	Mitochondrial O_2_^•−^ levels
JC1 5 µg/mL	488	520/590	Mitochondrial membrane potential

The fluorescence values of Fluo-4-, H_2_-DCFDA- or MitoSox RED-loaded cells are expressed as the means (±standard errors of the means (SEM)) of f/cell number, where f is the fluorescence value acquired, which was then normalized to the number of cells in the well. Fluorescence values of JC1-loaded cells are expressed as the means ( ± SEM) of the red/green fluorescence ratio, which depends on the mitochondrial membrane potential^60^. For each experimental condition, eight repetitions were performed in three independent experiments.

### Statistical analyses

Experimental values are expressed as means ± SEM. Statistical significance was assessed using Student’s t-tests with Prism5 software (GraphPad, San Diego, CA, USA). P values < 0.05 were considered statistically significant.

### Data availability statement

The authors confirm that all data supporting the findings are available without restriction.

## Electronic supplementary material

Supplementary Fig. S1 and S2

## References

[1] Zhang LF (2013). Region-specific vascular remodeling and its prevention by artificial gravity in weightless environment. Eur J Appl Physiol.

[2] Norsk P (2005). Cardiovascular and fluid volume control in humans in space. Curr Pharm Biotechnol.

[3] Stein, T. P. Weight, muscle and bone loss during space flight: another perspective. *Eur J Appl Physiol* **113**, 2171–2181, https://doi.org/10.1007/s00421-012-2548-9 (2013). 10.1007/s00421-012-2548-9 23192310

[4] Narici MV, de Boer MD (2011). Disuse of the musculo-skeletal system in space and on earth. Eur J Appl Physiol.

[5] Moore ST (2003). Ocular and perceptual responses to linear acceleration in microgravity: alterations in otolith function on the COSMOS and Neurolab flights. J Vestib Res.

[6] Mandsager KT, Robertson D, Diedrich A (2015). The function of the autonomic nervous system during spaceflight. Clin Auton Res.

[7] Macho L (2001). Endocrine responses to space flights. J Gravit Physiol.

[8] Drudi L, Grenon SM (2014). Women’s health in spaceflight. Aviat Space Environ Med.

[9] Jones JA, Jennings R, Pietryzk R, Ciftcioglu N, Stepaniak P (2005). Genitourinary issues during spaceflight: a review. Int J Impot Res.

[10] Macho L, Kvetnansky R, Fickova M, Popova IA, Grigoriev A (2001). Effects of exposure to space flight on endocrine regulations in experimental animals. Endocr Regul.

[11] Ronca AE (2014). Effects of sex and gender on adaptations to space: reproductive health. J Womens Health (Larchmt).

[12] Di Agostino S, Botti F, Di Carlo A, Sette C, Geremia R (2004). Meiotic progression of isolated mouse spermatocytes under simulated microgravity. Reproduction.

[13] Pellegrini M (2010). Microgravity promotes differentiation and meiotic entry of postnatal mouse male germ cells. PLoS One.

[14] Ricci G, Catizone A, Esposito R, Galdieri M (2004). Microgravity effect on testicular functions. J Gravit Physiol.

[15] Strollo F (2004). Microgravity-induced alterations in cultured testicular cells. J Gravit Physiol.

[16] Strollo F (1998). The effect of microgravity on testicular androgen secretion. Aviat Space Environ Med.

[17] Rijlaarsdam MA, Looijenga LH (2014). An oncofetal and developmental perspective on testicular germ cell cancer. Semin Cancer Biol.

[18] Young JC (2011). TCam-2 seminoma cell line exhibits characteristic foetal germ cell responses to TGF-beta ligands and retinoic acid. Int J Androl.

[19] Mizuno Y, Gotoh A, Kamidono S, Kitazawa S (1993). [Establishment and characterization of a new human testicular germ cell tumor cell line (TCam-2)]. Nihon Hinyokika Gakkai Zasshi.

[20] Nettersheim D (2013). Analysis of TET expression/activity and 5mC oxidation during normal and malignant germ cell development. PLoS One.

[21] Esposito F (2012). Thehigh-mobility group A1-estrogen receptor beta nuclear interaction is impaired in human testicular seminomas. J Cell Physiol.

[22] Eppelmann U (2013). Raman microspectroscopic discrimination of TCam-2 cultures reveals the presence of two sub-populations of cells. Cell Tissue Res.

[23] Nettersheim D, Gillis A, Biermann K, Looijenga LH, Schorle H (2011). The seminoma cell line TCam-2 is sensitive to HDAC inhibitor depsipeptide but tolerates various other chemotherapeutic drugs and loss of NANOG expression. Genes Chromosomes Cancer.

[24] Ferranti F (2013). TCam-2 seminoma cells exposed to egg-derived microenvironment modify their shape, adhesive pattern and migratory behaviour: a molecular and morphometric analysis. PLoS One.

[25] Nettersheim D, Gillis AJ, Looijenga LH, Schorle H (2011). TGF-beta1, EGF and FGF4 synergistically induce differentiation of the seminoma cell line TCam-2 into a cell type resembling mixed non-seminoma. Int J Androl.

[26] Ferranti F (2014). Cytoskeleton modifications and autophagy induction in TCam-2 seminoma cells exposed to simulated microgravity. Biomed Res Int.

[27] Kristensen DM (2008). Origin of pluripotent germ cell tumours: the role of microenvironment during embryonic development. Mol Cell Endocrinol.

[28] Looijenga LH, Van Agthoven T, Biermann K (2013). Development of malignant germ cells - the genvironmental hypothesis. Int J Dev Biol.

[29] Nettersheim D, Schorle H (2017). The plasticity of germ cell cancers and its dependence on the cellular microenvironment. J Cell Mol Med.

[30] Nettersheim D (2012). Establishment of a versatile seminoma model indicates cellular plasticity of germ cell tumor cells. Genes Chromosomes Cancer.

[31] Van Hooser A, Goodrich DW, Allis CD, Brinkley BR, Mancini MA (1998). Histone H3 phosphorylation is required for the initiation, but not maintenance, of mammalian chromosome condensation. J Cell Sci.

[32] Cirelli E (2017). Effect Of Microgravity On Aromatase Expression In Sertoli Cells. Sci Rep.

[33] Nowacki D, Klinger FG, Mazur G, De Felici M (2015). Effect of Culture in Simulated Microgravity on the Development of Mouse Embryonic Testes. Adv Clin Exp Med.

[34] Zhang X (2014). Mouse undifferentiated spermatogonial stem cells cultured as aggregates under simulated microgravity. Andrologia.

[35] Dai ZQ, Wang R, Ling SK, Wan YM, Li YH (2007). Simulated microgravity inhibits the proliferation and osteogenesis of rat bone marrow mesenchymal stem cells. Cell Prolif.

[36] Sun L (2008). Simulated microgravity alters multipotential differentiation of rat mesenchymal stem cells in association with reduced telomerase activity. Acta Astronaut.

[37] Plett PA, Abonour R, Frankovitz SM, Orschell CM (2004). Impact of modeled microgravity on migration, differentiation, and cell cycle control of primitive human hematopoietic progenitor cells. Exp Hematol.

[38] Chung JH (2016). Simulated Microgravity Effects on Nonsmall Cell Lung Cancer Cell Proliferation and Migration. Aerosp Med Hum Perform.

[39] Ma X (2014). Differential gene expression profile and altered cytokine secretion of thyroid cancer cells in space. FASEB J.

[40] Testa F (2014). Fractal analysis of shape changes in murine osteoblasts cultured under simulated microgravity. Rend. Fis. Acc. Lincei.

[41] Masiello MG (2014). Phenotypic switch induced by simulated microgravity on MDA-MB-231 breast cancer cells. Biomed Res Int.

[42] Ricci G, Esposito R, Catizone A, Galdieri M (2008). Direct effects of microgravity on testicular function: analysis of hystological, molecular and physiologic parameters. J Endocrinol Invest.

[43] Albi, E. *et al*. Impact of Gravity on Thyroid Cells. *Int J Mol Sci* **18**, doi:10.3390/ijms18050972 (2017). 10.3390/ijms18050972 PMC5454885 28471415

[44] Arfat Y (2014). Physiological effects of microgravity on bone cells. Calcif Tissue Int.

[45] Crawford-Young SJ (2006). Effects of microgravity on cell cytoskeleton and embryogenesis. Int J Dev Biol.

[46] Maier JA, Cialdai F, Monici M, Morbidelli L (2015). The impact of microgravity and hypergravity on endothelial cells. Biomed Res Int.

[47] Uva BM (2005). Morpho-functional alterations in testicular and nervous cells submitted to modelled microgravity. J Endocrinol Invest.

[48] De Luca C (2009). Monitoring antioxidant defenses and free radical production in space-flight, aviation and railway engine operators, for the prevention and treatment of oxidative stress, immunological impairment, and pre-mature cell aging. Toxicol Ind Health.

[49] Kaufmann I (2011). Adenosine A2(A) receptor modulates the oxidative stress response of primed polymorphonuclear leukocytes after parabolic flight. Hum Immunol.

[50] Peng L (2015). NADPH Oxidase Accounts for Changes in Cerebrovascular Redox Status in Hindlimb Unweighting Rats. Biomed Environ Sci.

[51] Wang Y (2016). Effect of Prolonged Simulated Microgravity on Metabolic Proteins in Rat Hippocampus: Steps toward Safe Space Travel. J Proteome Res.

[52] Zhang R (2014). Simulated microgravity-induced mitochondrial dysfunction in rat cerebral arteries. FASEB J.

[53] Li N, An L, Hang H (2015). Increased sensitivity of DNA damage response-deficient cells to stimulated microgravity-induced DNA lesions. PLoS One.

[54] Qu L (2010). Protective effects of flavonoids against oxidative stress induced by simulated microgravity in SH-SY5Y cells. Neurochem Res.

[55] Wang J (2009). Simulated microgravity promotes cellular senescence via oxidant stress in rat PC12 cells. Neurochem Int.

[56] Gorlach A, Bertram K, Hudecova S, Krizanova O (2015). Calcium and ROS: A mutual interplay. Redox Biol.

[57] Jacobson J, Duchen MR (2004). Interplay between mitochondria and cellular calcium signalling. Mol Cell Biochem.

[58] Amaral A, Lourenco B, Marques M, Ramalho-Santos J (2013). Mitochondria functionality and sperm quality. Reproduction.

[59] Guarnieri S (2009). Extracellular guanosine and GTP promote expression of differentiation markers and induce S-phase cell-cycle arrest in human SH-SY5Y neuroblastoma cells. Int J Dev Neurosci.

[60] Morabito C (2010). Modulation of redox status and calcium handling by extremely low frequency electromagnetic fields in C2C12 muscle cells: A real-time, single-cell approach. Free Radic Biol Med.

