# Supplementary material for: Transient increases in intracellular calcium and reactive oxygen species levels in TCam-2 cells exposed to microgravity
Source: Sci Rep. 2017 Nov 15;7:15648. doi: 10.1038/s41598-017-15935-z (PMC5688167; doi:10.1038/s41598-017-15935-z)
Supplement: Supplementary file 1 — Supplementary Fig. S1 and S2 [file 41598_2017_15935_MOESM1_ESM.pdf]

Transient increases in intracellular calcium and reactive oxygen species levels in TCam-2 cells exposed to microgravity

C. Morabito, S. Guarnieri, A. Catizone, C. Schiraldi, G. Ricci and M.A. Mariggiò

Supplementary Fig. S1

a b

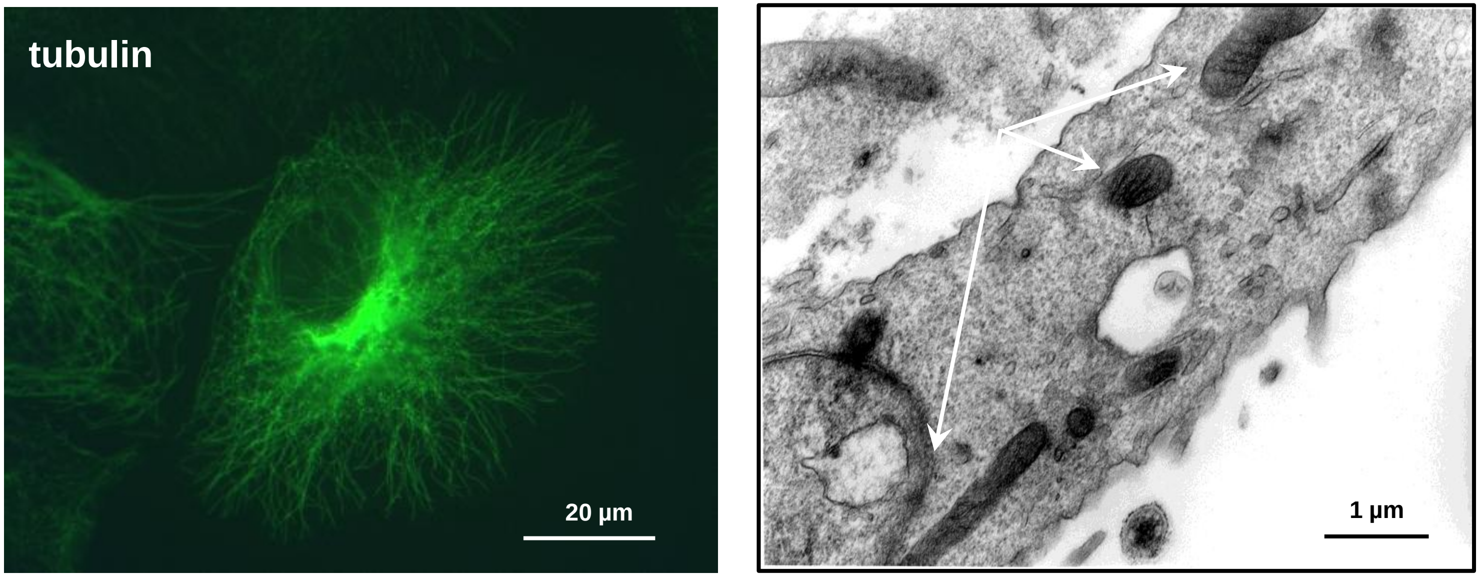

Phenotypic rescue of TCam-2 cells after 48 hours of simulated microgravity exposure. a. Representative α-tubulin immunofluorescence on TCam-2 cells exposed to simulated microgravity for 48 hours. b. TEM analysis of TCam-2 cell ultrastructure, after 48 hours of simulated microgravity exposure. The white arrows indicate the mitochondria.

Supplementary Fig. S2

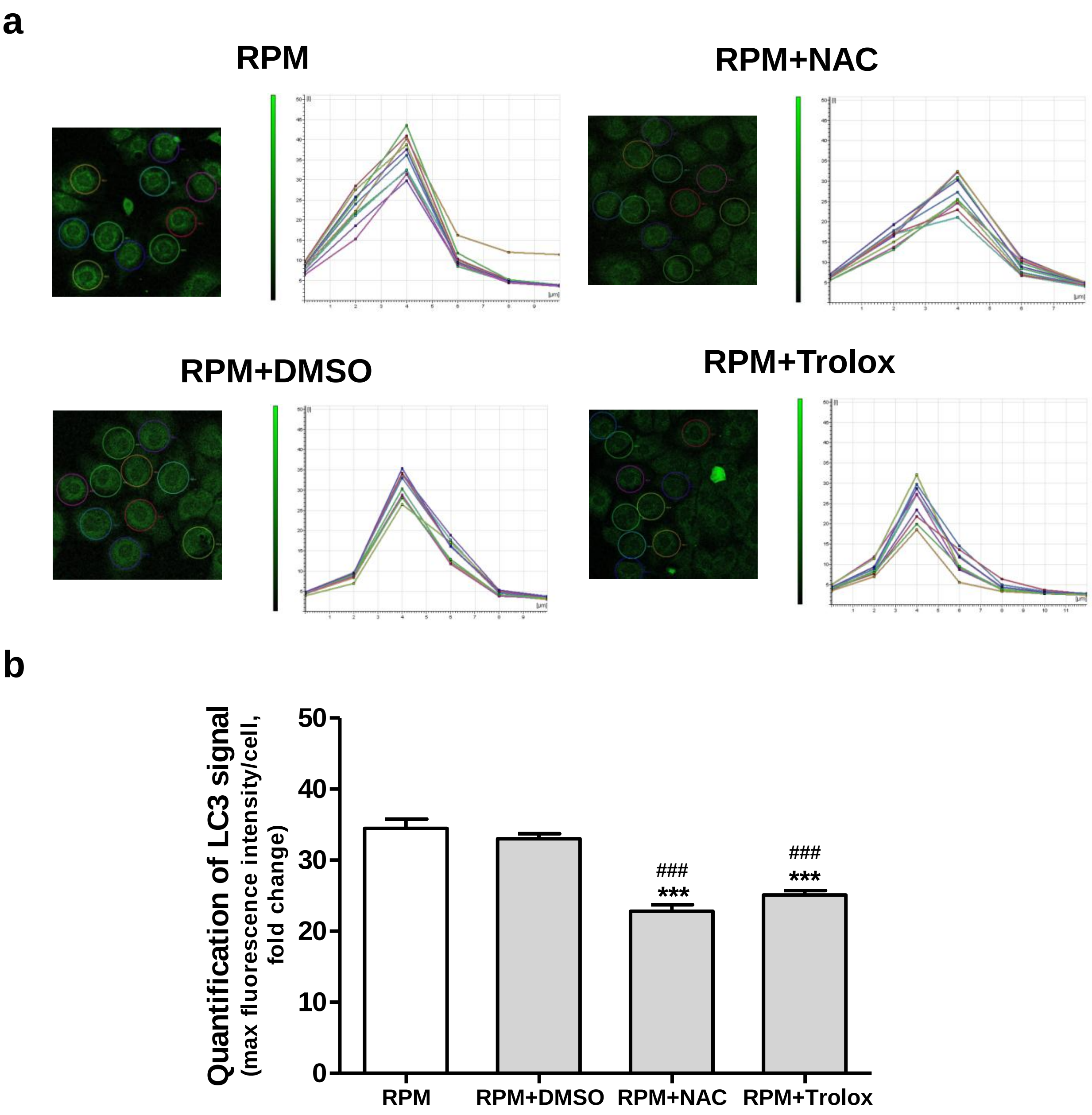

Antioxidants partly counteract the microgravity-induced cell autophagy. a. Representative analysis of LC3 fluorescence presented as “maximum of amplitude” on specific Region of Interest (ROI) of cells grew on RPM in growth medium alone (RPM) or in presence of NAC or Trolox (RPM+NAC or RPM+Trolox respectively). The sample RPM+DMSO were cells grown on RPM in growth medium containing DMSO that is the solution in which Trolox was solubilized. This analysis was performed on the "maximum intensity projection" images of stack profiles, using the Leica Confocal Software for quantitative analysis. b. A graphical representation of the “maximum of amplitude” values shown in a. The values are presented as means±SEM of 30 randomly selected ROIs/sample. *** $p< 0.001$ compared with the corresponding Ctr; ### $p< 0.001$ compared with the RPM+DMSO.
